# Strain-specific differences in *Neisseria gonorrhoeae *associated with the phase variable gene repertoire

**DOI:** 10.1186/1471-2180-5-21

**Published:** 2005-04-27

**Authors:** Philip W Jordan, Lori AS Snyder, Nigel J Saunders

**Affiliations:** 1 Bacterial Pathogenesis and Functional Genomics Group, The Sir William Dunn School of Pathology, University of Oxford, South Parks Road, Oxford, OX1 3RE, UK

## Abstract

**Background:**

There are several differences associated with the behaviour of the four main experimental *Neisseria gonorrhoeae *strains, FA1090, FA19, MS11, and F62. Although there is data concerning the gene complements of these strains, the reasons for the behavioural differences are currently unknown. Phase variation is a mechanism that occurs commonly within the *Neisseria *spp. and leads to switching of genes ON and OFF. This mechanism may provide a means for strains to express different combinations of genes, and differences in the strain-specific repertoire of phase variable genes may underlie the strain differences.

**Results:**

By genome comparison of the four publicly available neisserial genomes a revised list of 64 genes was created that have the potential to be phase variable in *N. gonorrhoeae*, excluding the *opa *and *pilC *genes. Amplification and sequencing of the repeat-containing regions of these genes allowed determination of the presence of the potentially unstable repeats and the ON/OFF expression state of these genes. 35 of the 64 genes show differences in the composition or length of the repeats, of which 28 are likely to be associated with phase variation. Two genes were expressed differentially between strains causing disseminated infection and uncomplicated gonorrhoea. Further study of one of these in a range of clinical isolates showed this association to be due to sample size and is not maintained in a larger sample.

**Conclusion:**

The results provide us with more evidence as to which genes identified through comparative genomics are indeed phase variable. The study indicates that there are large differences between these four *N. gonorrhoeae *strains in terms of gene expression during *in vitro *growth. It does not, however, identify any clear patterns by which previously reported behavioural differences can be correlated with the phase variable gene repertoire.

## Background

*Neisseria gonorrhoeae *is the causative agent of the sexually transmitted disease gonorrhoea. In the male this is typically associated with a purulent discharge from the urethra. However, in women, infection of the cervix is often asymptomatic while gonococcal spread up the urinary tract or invasion across the epithelial layers can cause additional complications such as pelvic inflammatory disease (PID) and disseminated gonococcal infection (DGI). Depending on geographical location, between 0.1%–3% of cases of uncomplicated gonorrhoeae (UG) disseminate and cause DGI [1-3]. The mechanisms that allow some strains to invade and cause DGI are not well understood.

Many attempts have been made to correlate disease phenotype with the genetic characteristics of isolates from DGI and UG. Certain phenotypes have been associated with DGI, including the arginine, hypoxanthine, and uracil (AHU) auxotype in which AHU^- ^strains tend to be more invasive [4,5]. Serum resistance, and therefore the ability to invade successfully, is associated with porin serotype 1A, via its interaction with C4 binding protein [6] as well as a role in the invasion of epithelial cells [7]. There is also a correlation between the presence of a combination of *atlA *and the *traG *variant, *sac*-4, and the DGI phenotype. These are thought to be a serum resistance locus and a cytotoxin that are located within the Gonococcal Genetic Island [8]. Addressing this issue clearly is difficult because it is possible that isolates capable of causing disseminated infection will be isolated from uncomplicated infections, and the converse may occur in hosts that are unable to produce a satisfactory immunological defence.

There are four experimental strains of *N. gonorrhoeae *commonly used in the laboratory. Strain FA1090, the only gonococcal strain for which a genome sequence is currently available , was isolated from the cervix of a patient with DGI. Strain FA19 was isolated from a patient with both UG and DGI. Strains F62 and MS11, both of which were isolated from patients with UG, are also frequently used. Several differences have been reported between these strains.

These include the ability to acquire iron from lactoferrin due to expression of lactoferrin binding protein (Lbp). A competitive advantage associated with this receptor in male urogenitary tract infection has been reported, however the absence of this gene from approximately half of gonococcal strains suggests that there may also be an advantage to its absence [9]. Other differences include an elevated ICAM-1 response generated in epithelial cell lines upon exposure to strain FA1090 as compared to strain MS11 [10]. One of the largest differences is the presence of the Gonococcal Genetic Island in strain MS11 [8] and strain FA19, but not the other two strains [11]. Microarray studies have revealed that there is little difference in the gene complements of these four strains. Indeed, these studies have shown that strain F62 contains all of the genes encoded by the FA1090 genome [11]. What comparative gene hybridization studies cannot tell us is whether there are small differences in the gene sequences, including frame-shifts or base substitutions, and whether there are differences in the phase variable gene repertoire.

Phase variation is a process employed by many bacteria to reversibly control gene expression. It is a switching mechanism that allows the gene to be reversibly turned ON or OFF. One difference between this and other regulatory mechanisms is that it does not occur in response to a stimulus but instead is mediated by changes to the DNA, which can occur during replication. There are many mechanisms for phase variation including inversions and transposon movement (for reviews see [12,13]), however, the most common mechanism within the *Neisseria *spp. is via a slippage mechanism in which the length of a simple sequence repeat changes during replication [14]. Within the coding sequence, changes in this repeat can cause frame-shifting and generation of truncated reading frames. Upstream of the coding sequences changes in repeat length can also alter the expression level of the genes by changing the efficiency with which RNA polymerase or other transcription factors bind to promoter components.

Due to the potential phase variation has to alter the expression profiles of a bacterium, this is one of the next logical areas to investigate to identify differences between the strains of *N. gonorrhoeae *that might account for differing behaviour. To achieve this a revised list of all of the potential phase variable genes, excluding *opa *and *pilC *genes, in *N. gonorrhoeae *was generated based upon the four publicly available neisserial genomes, *Neisseria meningitidis *serogroup A strain Z2491 [15], serogroup B strain MC58 [16], serogroup C strain FAM18 (available at ), and *N. gonorrhoeae *strain FA1090 (available at ). The presence of the potentially variable repeat and the expression status of each of these genes was then determined and compared between the four *N. gonorrhoeae *strains.

## Results and Discussion

### A revised list of potential phase variable genes in *Neisseria gonorrhoeae*

Potential phase variable genes were predicted through identification of simple sequence repeats within coding or promoter regions, the alteration in the length of which would alter the expression of the associated protein. Genes that are common to *N. gonorrhoeae *and different strains of *N. meningitidis *tend to be highly conserved, with typical sequence identities of greater than 90%. There is sufficient similarity between them for differences in the length of repeats, with the potential to disrupt reading frames, to increase the quality of phase variable gene predictions. In the *Neisseria *spp. the phase variable repertoire has been previously defined through three-way genome comparison to identify potential phase variation associated simple sequence repeats [17]. Based on the repeat tract length, the presence of the gene, the presence of variations in the repeat tract length, and the location of the repeat, each of the previously identified potential phase variable genes was scored for the probability of being phase variable. Using the additional genome sequence of *N. meningitidis *strain FAM18, the predicted phase variable gene repertoire was re-assessed through four-way genome sequence comparison (data not presented), using the same method as previously described [17]. In this way, those potential phase variable genes that are common between the four genome sequences could be evaluated in light of variation in the presence and length of the repeat tract associated with the genes. This analysis identified 64 repeats in *N. gonorrhoeae *that had the potential to mediate phase variation (Table 1), excluding the *opa *and *pilC *genes. These were investigated in *N. gonorrhoeae *strains FA1090, F62, FA19, and MS11. The *opa *and *pilC *genes were not included in this study because these genes are established as phase variable, to change very rapidly during culture, and do not have sufficiently distinct priming sites in the multiple alleles for amplification. Therefore analysis of these genes would not be possible, and it would not be possible to interpret the meaning of any observed changes.

### Repeat length changes

Repeat length and composition were compared between the four gonococcal strains and differences were observed in 35 of the 64 (55%) repeats studied (Table 2), although no sequence was obtained for the repeat in XNG1511. The variation can be seen in two forms; either as variation of the repeat length, or sequence composition, between the four strains of *N. gonorrhoeae *as determined on the basis of the predominant population following sequencing, for example XNG0412 in which strain FA1090 contains a (G)7 repeat tract while strain FA19 contains a (G)6 repeat tract. In addition, although PCR can generate variation in repeat numbers leading to mixed populations in sequencing for templates of homopolymeric tracts of (C or G)11 or greater, when variation is seen in shorter homopolymeric tracts, or in repeats composed of longer repeated motifs, this can be taken to indicate that variation has occurred *in vivo *during culture. This has been established in a previous study in *H. pylori *using a similar amplification and sequencing strategy [18]. Observed changes in these repeat tracts provide additional evidence of phase variation, and is indicated by 'plate' in Table 2. For example, XNG0080 in strain FA19 is identified as having a (C)10 tract in Table 2, but this is the predominant population and in fact the population contains (C)8-(C)10 tracts in this gene. 12 of the 36 variable repeats show this form of instability during culture.

The threshold at which variation is observed in repeat length for *N. gonorrhoeae *appears to be in close agreement with that used to identify genes that had the potential to be phase variable in *Neisseria *spp. Variation is seen in re peats of poly-G/C tracts ≥ 7 nucleotides (nt), and poly-A/T tracts of ≥ 9 nt, while tetramers, pentamers, and heptamers show variation between strains even at low copy numbers of the repeat (differences between strains are observed in most of these repeats with only 2–4 copies of the repeat present e.g. the (CCCAA) repeat in XNG0520). There were no differences observed in either of the dinucleotide repeat tracts. G/C repeats ≥ 9 nt, tetramers, and pentamers (of all observed sizes) are referred to as variable copy number repeats subsequently because they tend to be unstable during *in vitro *culture. Therefore, when a variable copy number repeat shows no variation in the sequence data the associated ON or OFF phenotype is likely to be adaptive for the *in vitro *culture conditions. For example a (C)11 repeat in the gene XNG1942/XNG1941 in strain MS11 is relatively stable *in vitro*. This gene contains a (C)6 repeat in strain FA1090, which shows the value of investigating the shorter repeats and identifying variation based upon this in other strains.

Of the repeats previously experimentally determined to be phase variable (candidacy of 'K' in Table 1), nine of the ten genes show variation in the repeat including *fetA *(XNG1989) and *lgtA *(XNG2045). Variation in the *hmbR *repeat is not seen as the tract is interrupted by base substitutions and indeed the gene is degenerate (i.e. contains multiple frameshifts and/or in frame termination codons) in all four strains. *hmbR *was previously reported to be degenerate in *N. gonorrhoeae *strain MS11 [19]. In some genes predicted from sequence analysis (including many encoding hypothetical proteins), but not previously demonstrated to be phase variable, differences in repeat length provide new evidence for the phase variability of these genes. Furthermore, in some repeats mixed populations are observed in the sequence data indicating that these genes are indeed being phase varied during *in vitro *culture e.g. *pglH *(XNG0080). In Table 2 these are marked as 'plate' in the column marked 'Differences in Repeat'.

### Repeat length stability in *N. gonorrhoeae *strain FA1090

The FA1090 strain used in this study has a separate passage history than that used for the genome sequence, yet the copy numbers of the repeats are highly consistent between those observed in this study and the genome sequence. The predominant populations of the variable copy number repeats are mostly the same as the genome sequence. Some variation in the copy number of tetramers and pentamers is observed, but these tend to be more variable in repeat length amongst all strains. There is only one low copy number repeat that differs in length compared to the genome sequence. This is in the coding sequence for methionine aminopeptidase (XNG1868) and the repeat is indicated as being (G)7 in the genome sequence but for all strains in this study it was observed as (G)6, which is unlikely to be phase variable. Variation in expression of this gene is probably not favourable for cell survival, as it codes for an important protein involved in removing the N-terminal methionine from nascent peptides. It is thought therefore that this variation is probably due to an error in the genome sequence rather than phase variation, and is consistent with independent sequencing that observed a (C)6 repeat in strain FA1090 (L Snyder, unpublished).

### Repeat length differences between the unrelated strains

Amongst the four strains, there are differences in copy number or composition of the repeat in 55% of the genes studied. Not all of these differences are necessarily associated with changes in expression. Table 2 shows the repeat length and expression status of the gene and it can be seen that some repeats are variable but still maintain an OFF phenotype e.g. the type I restriction enzyme S (XNG0388). Other repeats are associated with genes that are in fact degenerate, e.g. *hmbR *(XNG1216), *aspA *(XNG0986), and *porA *(XNG0832), an association that had been previously described [14]. Not all of the observed strain differences are due to repeat variation as in genes that are irreversibly switched ON or OFF. For *lbpA *(XNG0245), a gene that is highly variable in *N. meningitidis*, there is no evidence of phase variation in the gonococcus and what is seen instead is expression or absence due to a deletion in the N-terminus of the gene in both strains FA1090 and F62. In approximately 45% of isolates this deletion prevents expression of the Lbp receptor. It is proposed that there may be a selective advantage for non-expression of this gene over strains with the ability to extract iron from lactoferrin [9].

The potential expression state of each gene was then examined with respect to variation in the repeat length and frameshifts in the gene. These core experimental strains have been passaged repeatedly under *in vitro *laboratory conditions. Therefore, the expression state of the phase variable repertoire *in vitro *may not reflect the phenotypes expressed during infection. The expression state of strain FA1090 has, however, shown considerable stability through different passage histories, and the phenotypes in the other strains may also be similarly stable and different. Indeed, theoretical models indicate that in the absence of selection that favours more fit phenotypes, phase varied phenotypes and their associated repeats will tend to be stable [20]. There will still be random changes between equally fit alternatives, but the net rate of change within the population will be substantially slower than the changes that occur in the presence of selective conditions. Observed differences may well account for at least some of the reported differences in strain behaviour, even if the phase variable repertoires are similar.

The genes that contain repeats associated with differences between the strains are not evenly distributed among the different functional groups of genes investigated, with the genes for surface sugar biosynthesis (lipopolysaccharide (LPS) and pilin glycosylation) and restriction modification systems being the most variable in expression and repeat copy number.

It is known that there are four phase variable genes involved in LPS biosynthesis in *N. gonorrhoeae*, *lgtA*, *lgtC*, *lgtD*, and *lgtG*, and repeat length variation is observed in all these genes in this study. When these genes switch they can produce a diverse range of surface structures even within a single strain. *In vivo *certain LPS structures have been shown to provide a mechanism for serum resistance because they can bind CMP-NANA and this allows resistance to the antibody-mediated arm of the complement cascade [21].

The pilin glycosylation enzymes attach sugars to the pilus of the *Neisseria *spp. and the four studied here all show evidence of phase variation, with *in vitro *instability. Phase variation has previously been seen in *pgtA/pglA *(XNG1644), *pglE *(XNG0189), *pglG *(XNG0081) and *pglH *(XNG0080) [22-24]. The role of glycosylation is unclear, but as the pilus is a surface-exposed structure glycosylation may have a role in masking immunogenic parts of the pilus. Indeed it has been reported that glycosylation may inhibit complement-mediated lysis due to its ability to bind anti-gal antibodies [25]. It has been seen that galactosidase treatment of pili markedly reduces attachment of the pilus to host cells suggesting an importance of the galactose residues for attachment [26]. The role of phase variation in the genes that produce the saccharide is unclear, although it is possible that different sugar forms may be associated with different properties with respect to attachment and colonisation.

*N. meningitidis *contains four uptake systems for iron that are phase variable. These are the lactoferrin uptake system LbpAB, the haemoglobin uptake systems HmbR and HpuAB, and the siderophore receptor FetA. In the *N. gonorrhoeae *strain FA1090 genome sequence *hmbR *is degenerate due to a premature frameshift, and this study shows that the *hmbR *gene is not intact in any of the four gonococcal strains. The *lbpA *gene contains a deletion in strains FA1090 and F62, but is present and intact in strains FA19 and MS11 but is not phase variable. *fetA *and *hpuA *are both present and contain phase variable tracts that exhibit variation during *in vitro *culture.

### Genes with unassigned functions

13 of the 23 hypothetical genes show differences in their repeat lengths or composition. XNG0663 is a hypothetical protein that has limited homology to Curli-like repeat regions. These Curli-like repeats are found in *Escherichia coli *and are associated with adhesion to many different host cell proteins including MHC class I [27], and fibronectin [28]. They are also seen in *Salmonella enterica *[29] and *Porphyromonas gingivalis*. XNG0663 does not contain the same variable repeat as the homologous gene in the meningococcus as the poly-C repeat is interrupted by a T residue. It does, however, have a poly-T tract in which T(7) is associated with expression; but it is unlikely that this would be phase variable due to its length. The gene therefore appears to be fixed ON in strains F62 and MS11 and OFF in strains FA1090 and FA19.

Due to its apparent association with strains that cause DGI rather than UG the expression status of XNG0663 was determined in several clinical isolates of *N. gonorrhoeae*. In a study of 20 strains isolated from different types of infection it is apparent that the gene is present and turned ON in the majority of strains and that the suggested association with DGI is probably due to the initial small sample size. These results are shown in table 3.

## Conclusion

This study extends the number of *N. gonorrhoeae *phase variable genes for which there is direct or indirect evidence of repeat length changes from ten to 28, in addition to the *pilC *and *opa *genes. Based upon the four most commonly used experimental strains no clear association between the presence of phase variable versions of genes or their phenotypes and invasive potential could be identified, other than the previously described association with *pgtA *[30]. An apparent association with XNG0663 was discounted following analysis of a larger collection of strains.

Phase varied phenotypes appear to be relatively stable in *in vitro *culture. The differences in the expressed genes (rather than their ability to phase vary), and combinations of phase varied genes may still play a role in determining the outcome of infection. A recent study shows that during infection the colonisation fitness of *H. pylori *correlates strongly with a need to phase vary fewer genes [31]. It may be that the early behaviour and subsequent outcome of neisserial infection is also influenced by the phenotypes of inoculating populations.

## Methods

### Whole genome analysis

The complete genome sequences of *N. meningitidis *serogroup A strain Z2491 [15], serogroup B strain MC58 [16], and serogroup C strain FAM18 (Sanger Institute; ) and *N. gonorrhoeae *strain FA1090 (available at ) were analysed using previously described whole-genome analysis methodology [14,17,32], using an ACEDB graphical interface [33]. Briefly, repeats composed of perfect repeats with motifs of 1–10 bases were identified using ARRAYFINDER [34]. All repeats were displayed in their sequence contexts with respect to ORFs and termination codons using the tools within ACEDB, and their neisserial protein and nucleotide homologies. These complete genome sequence databases were then analyzed for simple DNA repeats within their sequence contexts to determine the repertoire of putative phase variable genes. Homopolymeric tracts of greater than 6 Gs or Cs, and greater than 8 As or Ts, were each investigated and repeats below these thresholds when associated with a frameshift. Other repeats composed of ≥ 4 copies of dinucleotides and ≥ 3 copies of tetramer and longer motifs were also investigated. All repeats were analyzed to interpret the significance of the repeat on the basis of sequence context and the potential effect of length variation on the expression of the associated reading frames.

The selected genes addressed in this study are listed in Table 1 with an indication of their likelihood of phase variation based upon sequence analysis and previous publications. In this Table K indicates a gene known and reported to be phase variable from previous studies. Strong candidates (S) include those in which the tract length differs in different strains, but has not yet been shown to vary within a single strain. Strong candidates also include genes with tract lengths similar in composition to those that have been seen to vary in other genes, particularly if this is the source of a frame-shift. For example, the adhesin XNG1371 is not found in the meningococcal genome sequences, but contains a (CAAG)20 tract; similar tracts being seen in *virG*, *vapA*, and NMB1507, the lengths of which differ between strains. Further, the homopolymeric tracts in the gonococcal genome specific hypothetical genes XNG0470, XNG0473, XNG0503a, XNG1000, XNG1207a, XNG1511, and XNG1577 are each the sole source of a frame-shift mutation within the coding region and are of lengths that have been seen to mediate phase variation in other genes.

Moderate candidates (M) are those in which the tract length is typical of a phase variable gene in one or more sequences, but for which there is no evidence of tract length differences with other strains, largely due to the absence of the gene or the absence of the repeat tract in other strains. When present, the repeat is either not associated with a frame-shift, as are those strain-specific genes that have been identified as strong candidates for phase variation, or the frame-shift is present in all strains assessed and/or the frame-shift associated repeat length is < 7 bp in a homopolymeric tract.

Low candidates (L) are those in which the repeat tract has not been seen to change. Also included in this category are those genes in which a short homopolymeric repeat tract (< 7 bp) is different between strains and results in a frame-shift mutation that may not be readily reversible. For example, the reduction in the homopolymeric repeat from an in-frame (C)7 to a frame-shifted (C)5 may have then lead to subsequent additional mutations in the gene, rendering it degenerate (e.g. XNG0158).

### Bacterial strains and growth conditions

The strains used are shown below in Table 4. Strains were grown on GC agar (Difco Laboratories) with the Kellogg supplement and ferric nitrate [35] at 37°C under 5% (v/v) CO2. Cultures were grown from frozen stocks, passaged once the following day to obtain an inoculum for semi-confluent growth, and DNA was prepared from young healthy colonies early the following morning.

### PCR amplification and sequencing

Chromosomal DNA extractions were performed using the Aquapure genomic DNA kit (BioRad). PCR from chromosomal DNA was performed using Hotstar *Taq *DNA Polymerase (Qiagen) according to the manufacturers' instructions. Primer pairs used are shown in Table 5. If amplification was not successful with primer pair 1 it was repeated with primer pair 2. Automated sequencing was done using ABI Prism^® ^BigDye™ Terminator Cycle Sequencing version 3.0 (Applied Biosystems), and was resolved on an ABI Prism^® ^3100 DNA Sequencer (Applied Biosystems).

### Bioinformatics

ACEDB[33] was used to analyze the complete genome sequences of the *Neisseria *spp., as described previously[17]. Sequences were read, edited and aligned using Seqlab (Wisconsin Package, Version 10.2, Genetics Computer Group, Madison, Wisc., USA) through the Sir William Dunn School of Pathology/WIMM Computational Biology Research Group (CBRG). Homology searches were performed using BLAST[36] against the EMBL databases, accessed through the CBRG.

## Authors' contributions

PWJ carried out the PCR, sequencing, sequence alignment, and drafted the manuscript. LASS generated the list of phase variable genes. NJS conceived of the study, and participated in its design and coordination and helped to draft the manuscript. All authors read and approved the final manuscript.

## Figures and Tables

**Table 1:** List of genes identified as potentially being phase variable in *N. gonorrhoeae *– excluding the *opa *and *pilC *genes.

**XNG# †**	**TRACT ‡**	CANDIDACY *	**FUNCTIONAL ROLE**	**GENE**	**ANNOTATED FUNCTION**
XNG0023	GGGGCGG -	L	Other		prolyl oligopeptidase family protein
XNG0060	C(G)6 -	S	Surface sugar biosynthesis	wbpC / pglI	putative LPS biosynthesis or pilin glycosylation protein
XNG0075	(C)7 -	L	Hypothetical protein		hypothetical protein
XNG0080	(C)10 -	S	Hypothetical protein	pglH	pilin glycosylation protein
XNG0081	A(C)7 -	S	Surface sugar biosynthesis	pglG	pilin glycosylation protein
XNG0112	(A)6 p	S	Hypothetical protein		hypothetical protein
XNG0115	(C)4 -	L	Other	cvaA	putative colicin V secretion protein
XNG0158	(C)5 degenerate	L	Hypothetical protein		hypothetical protein
XNG0188	AA(C)5 -	L	Other	potD-2	spermidine/putrescine ABC transporter,
XNG0189	(CAAACAC)4 -	K	Surface sugar biosynthesis	pglE	pilin glycosylation protein
XNG0245	degenerate	S	Iron acquisition	lbpA	lactoferrin-binding protein A
XNG0388	(G)7 +	S	R-M Systems	hsdS||	type I restriction enzyme S protein
XNG0412	(G)7 +	S	Hypothetical protein		hypothetical protein
XNG0470	(G)7 +	S	Hypothetical protein		hypothetical protein ††
XNG0473	(T)9 +	S	Hypothetical protein		hypothetical protein
XNG0503a	(C)6A(C)9 +	S	Hypothetical protein		hypothetical protein
XNG0508	AACCGGCAAACA -	L / M	Hypothetical protein		hypothetical protein
XNG0520	(CCCAA)12 -	S	R-M Systems		type III restriction system methylase
XNG0552	(C)5 degenerate	L	Hypothetical protein		hypothetical protein
XNG0608	CCACACCC -	M	Other	nifS	aminotransferase
XNG0610	(G)7 -	L	Other	lldD	L-lactate dehydrogenase
XNG0612	(GCCA)37 -	S	R-M Systems		putative restriction system methylase
XNG0644	(A)9 -	L	R-M Systems		modification methylase NlaIV
XNG0663	CCCCTCC +	L	Hypothetical protein		hypothetical protein
XNG0703	(C)7 -	L	Other	dnaX	DNA polymerase III, subunits gamma and tau
XNG0714	GGAAGG -	L	Other	mobA	molybdopterin-guanine dinucleotide biosynthesis
XNG0832	(T)11C(G)6 p	K	Surface associated proteins	porA	outer membrane protein
XNG0887	(AAGC)4 -	S	Hypothetical protein		hypothetical protein
XNG0905	(AAGC)3 +	S	Surface associated proteins	virG	AIDA related protein
XNG0954	(C)7 -	L	Other	ppx	exopolyphosphatase
XNG0973	(C)6 -	L	Hypothetical protein		hypothetical protein
XNG0986	(C)6 degenerate	S	Other	aspA	putative serotype-1-specific antigen
XNG1000	(G)7 +	S	Hypothetical protein		hypothetical protein
XNG1041	piv-Nm1B inserted	S	Surface sugar biosynthesis		putative glycosyl transferase
XNG1207a	(G)8 +	S	Hypothetical protein		hypothetical protein **
XNG1216	GGCGGCCAA	K	Iron acquisition	hmbR	hemoglobin receptor
XNG1268	(AT)5 -	L	Other	fixP	cytochrome c oxidase, subunit III
XNG1281	G(A)7 -	S	Hypothetical protein		hypothetical protein
XNG1341	(CAAG)20	S	Surface associated proteins		adhesin
XNG1356	CCCTGCACC p	M	Other	pntA	NAD(P) transhydrogenase alpha subunit
XNG1384	TCCGCCC -	L	Other	amiC	N-acetylmuramoyl-L-alanine amidase
XNG1423	TGTGGGGG -	S	Other	dca	competence-associated protein
XNG1460	(C)7 -	L	Hypothetical protein		hypothetical protein
XNG1511	(G)8 +	S	Hypothetical protein		hypothetical protein
XNG1563	(AAGC)3 +	S	Surface associated proteins	vapA	virulence associated protein
XNG1577	(G)8 +	S	Hypothetical protein		hypothetical protein **
XNG1589	(C)4TCC -	L	Other	dinG	probable ATP-dependent helicase
XNG1644	(G)11 -	K	Surface sugar biosynthesis	pgtA	pilin glycosylation protein
XNG1733	(G)8 -	M	Hypothetical protein		hypothetical protein
XNG1740	(C)7 -	L	Other	rplK	50S ribosomal protein L11
XNG1788	(G)8 +	M	Hypothetical protein		hypothetical protein **
XNG1834	(C)8 -	M	Hypothetical protein		hypothetical protein
XNG1856	(TA)5 -	L	Hypothetical protein		hypothetical protein
XNG1858	(G)5 -	L	Surface associated proteins	mafA-3	putative adhesin
XNG1868	(C)7 +	L	Other	map	methionine aminopeptidase
XNG1934	(A)9 p	S	Other		putative lipoprotein
XNG1942/41	(C)6 +	S	Other		putative hydrolase
XNG1968	(C)11 -	K	Surface sugar biosynthesis	lgtG	lipopolysaccharide glycosyl transferase
XNG1989	(C)13 p	K	Iron acquisition	frpB / fetA	iron-regulated outer membrane protein
XNG2004	(G)9 +	K	Iron acquisition	hpuA	hemoglobin-haptoglobin utilization protein
XNG2045	(G)11 +	K	Surface sugar biosynthesis	lgtA	acto-N-neotetraose biosynthesis glycosyl transferase
XNG2047	(G)14 +	K	Surface sugar biosynthesis	lgtC	LPS biosynthesis protein
XNG2048	(G)14 -	K	Surface sugar biosynthesis	lgtD	LPS biosynthesis protein
XNG2061	(TTCC)3 -	L	Other	plsX	fatty acid-phospholipid synthesis protein

† CDS designation numbers come from our own annotation of the *N. gonorrhoeae *strain FA1090 genome sequence.

‡ The known or potential phase variable repeat tract from *N. gonorrhoeae *strain FA1090. The equivalent sequence is identified for genes without a repeat tract that are phase variable candidates from the meningococci. Following the sequence a - or + signifies if the CDS is in-frame or frame-shifted, respectively. Repeats in promoter or predicted promoter locations are indicated with a p. Degenerate indicates a gene with multiple frame-shifts or in-frame termination conditions.

* The predicted likelihood of phase variability. K indicates a known phase variable gene; S indicates a strong candidate for phase variation; M indicates a moderate candidate; L indicates a low candidate. See text for details of these thresholds.

†† XNG0470 is a homologue of XNG1014, and XNG1513 which have a (G)6 and are not frame-shifted.

** XNG1207a, XNG1577, and XNG1788 are homologues of each other.

**Table 2:** Repeat lengths for each of the genes.

**Functional Role**	**Gene**	**Annotated Function**	FA1090*	FA19*	F62*	MS11*	**Differences in repeat‡**
**Surface Associated Proteins**	XNG0832	Outer membrane protein	(T)11C(G)6 p	(T)8C(G)8 p	(T)11C(G)6 p	(T)9C(G)6 p	Plate
	XNG0905	AIDA related protein	(AAGC)3	(AAGC)2	(AAGC)3	(AAGC)3	Strains
	XNG1341	Adhesin	(CAAG)20	(CAAG)13	(CAAG)9	(CAAG)6	Plate
	XNG1563	Virulence associated protein	(AAGC)3	(AAGC)14	(AAGC)26	(AAGC)3	Plate
	XNG1858	Putative adhesin	(G)5	(G)5	(G)5	(G)5	

**Surface Sugar Biosynthesis**	XNG0060	LPS synthesis/pilin glycosylation	C(G)6	C(G)6	C(G)6	C(G)6	
	XNG0080	Pilin glycosylation protein	(C)10	(C)10	(C)10	Gene not present†	Plate
	XNG0081	Pilin glycosylation protein	A(C)7	A(C) 9	A(C)7 -	Gene not present†	Plate
	XNG0189	Pilin glycosylation protein	(CAAACAC)4	(CAAACAC)6(CAAATAC)3	(CAAACAC)4	(CAAACAC)15(CAAATAC)2	Plate
	XNG1041	Putative glycosyl transferase	piv-Nm1B inserted‡‡	piv-Nm1B inserted‡‡	piv-Nm1B inserted‡‡	piv-Nm1B inserted‡‡	
	XNG1644	Pilin glycosylation protein	(G)11	(G)17	GGGAGCGG	GGGAGCGG	Strains
	XNG1968	LPS glycosyl transferase	(C)11	(C)10	(C)10	(C)12	Strains
	XNG2045	Glycosyl transferase	(G)11	(G)12A	(G)16	(G)12	Plate
	XNG2047	LPS biosynthesis protein	(G)14	(G)13	(G)10	(G)8	Strains
	XNG2048	LPS biosynthesis protein	(G)13	(G)13	(G)11	(G)13	Strains

**Iron Acquisition**	XNG0245	Lactoferrin-binding protein A	Gene dead††	(G)5CGG	Gene dead††	TGAAACGG	Strains
	XNG1216	Hemoglobin receptor	GGCGGCCAA	GGCGGCCAA	GGCGGCCAA	GGCGGCCAA	
	XNG1989	Iron-regulated OMP	(C)13 p	(C)12 p	T(C)10 p	(C)15 p	Plate
	XNG2004	Hb-haptoglobin utilization	(G)9	(G)9	(G)11	(G)9	Plate

**Restriction Modification Systems**	XNG0388	Type I restriction enzyme S	(G)7	(G)8	(G)8	(G)7	Strains
	XNG0520	Type III restriction methylase	(CCCAA)3	(CCCAA)2	(CCCAA)11	(CCCAA)8	Plate
	XNG0612	Putative methylase	(GCCA)11	No sequence**	No sequence**	(GCCA)22	Plate
	XNG0644	Modification methylase NlaIV	(A)9	(A)9	(A)9	(A)9	

**Other**	XNG0023	Prolyl oligopeptidase family	GGGGCGG	GGGGCGG	GGGGCGG	GGGGCGG	
	XNG0115	Putative colicin V secretion protein	(C)4	(C)4	(C)4	(C)4	
	XNG0188	ABC transporter component	AA(C)5	AA(C)5	AA(C)5	AA(C)5	
	XNG0608	Aminotransferase	CCACACCC	CCACACCC	CCACACCC	CCACACCC	
	XNG0610	L-lactate dehydrogenase	(G)7	(G)7	(G)7	(G)7	
	XNG0703	DNA polymerase III subunits	(C)7	(C)7	(C)7	(C)7	
	XNG0714	Biosynthesis protein	GGAAGG	GGAAGG	GGAAGG	GGAAGG	
	XNG0954	Exopolyphosphatase	(C)7	(C)7	(C)7	(C)7	
	XNG0986	Putative serotype-1-specific antigen	(C)6	(C)6	(C)6	(C)7	Strains
	XNG1268	Cytochrome c oxidase, subunit III	(AT)5	(AT)5	(AT)5	(AT)5	
	XNG1356	NAD(P) transhydrogenase subunit	CCCTGCACC p	CCCTGCACC p	CCCTGCACC p	CCCTGCACC p	
	XNG1384	N-acetylmuramoyl-L-alanine amidase	TCCGCCC	TCCGCCC	TCCGCCC	TCCGCCC	
	XNG1423	Competence-associated protein	TGTGGGGG	TGTGGGGG	TGTGGGGG	TGTGGGGG	
	XNG1589	Probable ATP-dependent helicase	(C)4TCC	(C)4TCC	(C)4TCC	(C)4TCC	
	XNG1740	50S ribosomal protein L11	(C)7	(C)7	(C)7	(C)7	
	XNG1868	Methionine aminopeptidase	(C)6	(C)6	(C)6	(C)6	Genome
	XNG1934	Putative lipoprotein	(A)9	(A)8 p	(A)8 p	(A)8 p	Strains
	XNG1942/41	Putative hydrolase	(C)6	CAAACCCC	(C)6	(C)11	Strains
	XNG2061	Fatty acid-phospholipid synthesis	(TTCC)3	(TTCC)3	(TTCC)3	(TTCC)3	

**Hypothetical Proteins**	XNG0075	Hypothetical protein	(C)7	(C)7	(C)7	(C)7	
	XNG0112	Hypothetical protein	(A)6 p	(A)6 p	(A)6 p	(A)6 p	
	XNG0158	Hypothetical protein	(C)5	(C)5	(C)5	(C)5	
	XNG0412	Hypothetical protein	(G)7	(G)6	(G)6	not the same gene***	Strains
	XNG0470	Hypothetical protein	(G)7T	(G)6	(G)6CC	(G)7T	Strains
	XNG0473	Hypothetical protein	(T)9	(T)8	(T)9		Strains
	XNG0503a	Hypothetical protein	(C)6A(C)9	(C)7A(C)4T(C)3	(C)6A(C)7	(C)6A(C)13	Plate
	XNG0508	Hypothetical protein	AACCGGCAAACA	AACCGGCAAACA	AACCGGCAAACA	AACCGGCAAACA	
	XNG0552	Hypothetical protein	(C)5	(C)5	(C)5	(C)4	Strains
	XNG0663	Hypothetical protein	CCCCTCC & T(7)	CCCCTCC & T(7)	CCCCTCC & T(6)	CCCCTCC & T(6)	Strains
	XNG0887	Hypothetical protein	(AAGC)4	(AAGC)15	(AAGC)20	(AAGC)7	Strains
	XNG0973	Hypothetical protein	(C)6	(C)6	(C)6	(C)6	
	XNG1000	Hypothetical protein	(G)7(T)4	(G)7(T)6	(G)8(T)4	GAGG(T)6	Strains
	XNG1207a	Hypothetical protein	(G)8TTGTT	(G)8T	(G)6TTGTT	(G)10TT	Strains
	XNG1281	Hypothetical protein	G(A)7	(A)8	G(A)7	G(A)7	Strains
	XNG1460	Hypothetical protein	(C)7	(C)7	(C)7	(C)7	
	XNG1511	Hypothetical protein	No sequence**	No sequence**	No sequence**	No sequence**	
	XNG1577	Hypothetical protein	GGA(G)8TTGTT	GGA(G)7T	(G)6TTGTT	GGA(G)4TTGTT	Strains
	XNG1733	Hypothetical protein	(G)8	(G)7	(G)10	(G)7	Plate
	XNG1788	Hypothetical protein	(G)8TTGTT	(G)6TTGTT	(G)6TTGTT	(G)12TT	Plate
	XNG1834	Hypothetical protein	(C)8	(C)8	(C)9	(C)8	Plate
	XNG1856	Hypothetical protein	(TA)5	(TA)5	(TA)5	(TA)5	

* A clear box indicates that the gene is expressed. A shaded box indicates that the gene is not expressed.

‡ Indicating variation in the repeat. 'Strains' indicates differences in the repeat between strains. 'Plate' indicates variation of the repeat during *in vitro *culture. 'Genome' indicates that there is variation from the genome sequence but no observed differences between strains.

† This gene is not present in this strain as seen by microarray comparative genome hybridisation [11].

‡‡ The site-specific recombinase piv-Nm1B has been inserted into this gene in all four strains

†† A large deletion is present in the N-terminus of this gene.

** Amplification and sequencing did not work.

*** Sequence analysis showed that the ORF present here had no homology to XNG0412 from strain FA1090.

**Table 3:** Clinical strains of *N. gonorrhoeae *and their expression of XNG0663.

Gonococcal Strain	Site of Isolation	Composition of poly-C tract	Composition of poly-T tract	Gene expression*
25527	Blood	CCCCTCC	(T)6	ON
25563	Blood	CCCCTCC	(T)6	ON
26034	Blood	CCCCTCC	(T)6	OFF
26241	Blood	CCCCTCC	(T)6	OFF
26593	Blood	CCCCTCC	(T)6	ON
25534	Joint fluid	CCCCTCC	(T)6	ON
25562	Joint fluid	CCCCTCC	(T)6	ON
26399	Joint fluid	CCCCTCC	(T)7	ON
26775	Joint fluid	CCCCTCC	(T)6	ON
27706	GC	CCCCTCC	(T)6	ON
27833	GC	CCCCTCC	(T)6	ON
27886	GC	CCCCTCC	(T)6	OFF
27921	GC	CCCCTCC	(T)6	ON
28197	GC	CCCCTCC	(T)6	ON
28252	GC	CCCCTCC	(T)6	ON
28386	GC	CCCCTCC	(T)7	ON

* Gene expression also depends on the presence or absence of an adenine residue between the two tracts. Therefore strains with the same poly-T repeat can be in the open reading frame or be frame-shifted.

**Table 4:** Gonococcal strains used in this study

STRAIN	SITE OF ISOLATION	ISOLATED FROM
		
25527	Blood	DGI
25534	Joint fluid	DGI
25562	Joint fluid	DGI
25563	Blood	DGI
26034	Blood	DGI
26241	Blood	DGI
26399	Joint fluid	DGI
26593	Blood	DGI
26775	Joint fluid	DGI
27706	GC	UG
27728	GC	UG
27806	GC	UG
27833	GC	UG
27886	GC	UG
27921	GC	UG
28197	GC	UG
28252	GC	UG
28386	GC	UG
28480	GC	UG
28539	Joint fluid	DGI
FA1090	Blood	DGI
FA19	Blood	DGI
F62	GC	UG
MS11	GC	UG

**Table 5:** Primers used for amplification in this study

**Gene**	**Forward Primer**	**Reverse Primer**
		
XNG0023-1	GTATTCGTCCGTCCAACTTGAACCG	GTGGATTTGGAAGCAGGGGAATTGG
XNG0060-1	TGGAAGGCAAGGAATACGTC	GCGGATTTCCACAAAAGAAA
XNG0075-1	CGATGGATACGACCCTGAAGC	CGTCCTGCTCGTCGAAATCC
XNG0080-1	GACTGCATGGCGTAAGAGTGG	GCCATCACCGCTTCGTCAAAC
XNG0081-1	CAAAAACGGCACGACAGAG	GCGATTTGACCGGCTACTT
XNG0112-1	CAAACCCAAACCGTATGC	CGTTCTTTGCCGAGAAAGTC
XNG0115-1	ATCCCGACAATTCCTGTCTG	CTATTTGCGCTTTCGACCTC
XNG0158-1	AGTACGGCAGTGGTTTGTCC	AACCTCACCCTCCACTTTCC
XNG0188-1	GCTTCAGACGGCATTTTCAC	AAATAGGGGACGGCGTATTC
XNG0189-1	TTGTGAATGACGGGTCAAAA	TCGACCCCGAATAATGTGAT
XNG0245-1	GTAACGGGCGGCTTTTACG	TATTCGATTTCGTTGATTGCG
XNG0388-1	TTCGCATAAAAATGAAATGTGG	CCCGGATAACCAAAACATCA
XNG0412-1	TATCTGTGGCACGAAGAACG	CTATCCCCTTCCATGCAATC
XNG0470-1	AATGTACGCCGGTCTGAAAG	ATATCCGCCACTTTTTGCAG
XNG0470-2	AAACCGCGCCTTCGAGTGCG	TTCAGGGCGTTGTCCAAATCG
XNG0473-1	GAAATCGGCTTGAGATACGG	AATCCCTGTTCCGGATTTTC
XNG0503a-1	CGCGTGTTGCCTAAAAACA	TCAAAACGTACTCCCCCTTG
XNG0508-1	TGTTGTTGAAGGCTTCGTTG	CTTCCGCATCCTGCAAAT
XNG0520-1	ATACGGCTACACCGTTTTCG	TCTTCACGCTGTTTGCGTAG
XNG0552-1	ATTCGAGTTGGCAAAACACC	GCGGGAATGTATCCTTGTTG
XNG0608-1	ATCAACATCGCCCAAATCAT	ATCAAACCGTTTTCCTGCAC
XNG0610-1	CAAGCGCAAAATGCCGCGTATG	TTGACCCAAAACCTGCAAATCGG
XNG0612-1	GTGGAAAAAACCGACCCGTCC	CATTGAACAAAGATTAGCCCATCATC
XNG0612-2	CAAACCGAATTAGCCCAGCC	TCGGTTTCTGTATTATACGG
XNG0644-1	CCCTGACCGTTATTTTGGAA	AGGTGCTCCATATCCATTGC
XNG0663-1	CCCCCACAGCAACATAGAAT	TTGGACTGGAGGCCATTTAC
XNG0703-1	TCTTGGAAAACGCCCAATAC	GGATTTGTTCGCCGCTTAT
XNG0714-1	CGCCAAACCGACCCACAAG	CGTCAAACTCGAATCTGACCG
XNG0832-1	GGCAAGGAAGAAGGGGATAC	CGTATAGGCGGACTTGCTGT
XNG0832-2	CGAAAACGCCGAATACGAAGC	GGCATCGCCAAACGGATTCGCA
XNG0887-1	ACCTGCAATACGCCTACACC	TGTCGTCCTGTGTGTTGAAA
XNG0905-1	GGCCATAGTTTCCGAAGGAT	TTAATCGGAAAAACCGAACG
XNG0905-2	CGATTTTTTCATTGAAGACCG	GGAGTGGTTTTTCTTCTTCCC
XNG0954-1	CCGCCGGTCTGGACGAAC	ACGCAGGATGAAGCCGCAGC
XNG0973-1	GCTTTCACACTCGTCAGCAC	TTTCGTTTCGTTCTGGGTTT
XNG0973-2	GTTTTTCAGCTTGTCCGAACC	ACGCTTCCGGCTTGACATGG
XNG0986-1	CGGCGTTTTAACAACAGTGA	TGGTTCCATCATGCGTATTC
XNG1000-1	GGAAACCCTGTTCCAAGACA	CGGGCGATGAGATGAAGTAT
XNG1041-1	GAAAAATACCTTCGCTGCTG	TGATTCCGGACTGAGATACG
XNG1207a-1	GCTTCAAAGAAACCGACAGG	CGCTCAAAGCCTGAAAAGTC
XNG1216-1	GACACGGTAGCCGGAAGTAA	AGCAGGGGCATAATTACACG
XNG1268-1	TGTCGTGCCTGAGTCCAATA	AACCAATCGCTATCGGTCAG
XNG1268-2	CATCCGTTCCAATGGGGTTCC	TCTTCGCCCAAAGCAGGACC
XNG1281-1	CAAAACCGCTCAAAGCCTAC	CTTTTGCTTCCCATGCTTGT
XNG1281-2	GACGCGGTCGTTAAAACTCCC	GAAGTCAAACAAGTCAACGACC
XNG1341-1	TCACGTCCTGCATACTCTCG	ATGCCAGATAGCCTTCTGCT
XNG1341-2	GCTCACCTCGGCATACTGCC	TTTTCGATGCTTCAGAACTTCC
XNG1356-1	TCGAATCCATATCGCTGACA	AAGCTGACGATGGTTTGACC
XNG1356-2	ATCCAACTCTCCGCGCATGG	GGTAGCCGCTGATGTTTGCC
XNG1384-1	ACACCTACACCCGCCTGAC	GCGTGGATGGAGACGAATAC
XNG1423-1	GCAATGGGAAACCGTAAACA	GCTTTGGCTTCGGTTGTATT
XNG1423-2	ACGACATCATCAACGACATCC	AACATTCCTTGATTAGACAGCC
XNG1460-1	TGCTGTGCAGTCAAAACCTC	TGTACATTTGACGGGCATCT
XNG1460-2	GGACGGCGGCAAATGGCTGG	TCAGTTCGGCGGTGCTGTGC
XNG1511-1	GGAGTACGCCATCGCCAGC	GCGATGCCGCAGGCAAGG
XNG1511-2	TGCGACTGCGCCAACGACG	GACGGCGTAGTTTTCAATCC
XNG1563-1	CCGCAAACTGTATTCCGAAC	CCGCTGGAGAAATAATGGAA
XNG1577-1	AAAGTATCCCTTCCGCCTGT	ATTGTCCGTTTTGAGCTTGC
XNG1589-1	GCCCGTATCGAACAAATCAG	GGAGTTTGGCGATGATGACT
XNG1644-1	GGCATTTTGATTGCCCTGTA	TAACTTGCATTCGCCATCAG
XNG1644-2	GTTTGGGATTCGCATTTACCC	GGAAGCCGTTGACCTTGTCG
XNG1733-1	TCCTTCAATTGAGCCAGAGC	GCAAATGTTGATGCGAAAGA
XNG1740-1	GTCAGCAGCAGTCAAATCAGGC	TAACGGGGTGGTTGAGGAGG
XNG1788-1	CGCGAATTGCTGGTAAAAAT	AAAGTATCCCTTCCGCCTGT
XNG1834-1	GATGCAGCCCTGATGAGTCGG	CCATCTCCACCAAAATCGCACC
XNG1856-1	TCGTCAAATTCCCAGTCAAA	CGGTAAAAGACGGCATCAAT
XNG1856-2	TGGGATTGGGTTAAAAATACC	GATTATAATCGTCAAATTCCC
XNG1858-1	ATATCGTCGTCTTCGGCATC	GCATTGGTTTTGGGTTTGAT
XNG1858-2	GGTCGAACAAGAACTTGTGG	TCCTTTGCTTACTTTATACGG
XNG1868-1	CCAAGACTTCGTGTTCCCATTG	CCGCAATCCAAGGAAGCATTATG
XNG1868RF-2	ACGGCATCATCATCAAAACC	AGACTTCGTGTTCCCATTGG
XNG1934-1	GCCGTCTGAATACACAGCAA	ACGCTGCTGATACGTTTGGT
XNG1934-2	GTCTGAAGGCTTGGGCTTGC	TTTCCAATTCGTCAAACTCG
XNG1942/XNG1941-1	ACACCCTATGCCGTCTGAAC	TTTGGTTTTTGCCAGTGTTG
XNG1968-1	GTGCAGAAGCCCAGTCCGAC	CCCATCTTGTCGATCAACGC
XNG1989-1	GGAATTGGGACGTGCCGTTG	CGATGACTTCGCCCATATCGG
XNG2004-1	ACGGTTATGGGGTCGGCGTAG	ACAGCAGGAAATCCCCGTCG
XNG2045-1	GTGCAAAACCTGCTGAAAAA	CCCAATTTGCTGACATCGTA
XNG2047-1	ACACATTCAAACACCGCCTG	TGGTACGGTCGAGGTAAAGC
XNG2048-1	CGACAAAGCGTATGCTTCAA	GACCAAGGCTTCGGGATAAT
XNG2061-1	ATGTCCGCCTGATTATGACC	CCTTCGATGGTTTTGAGCAT
